# Complete Genome Sequences of Five *Burkholderia* Strains with Biocontrol Activity against Various Lettuce Pathogens

**DOI:** 10.1128/mra.01120-21

**Published:** 2022-01-13

**Authors:** Adrien Biessy, Marie Ciotola, Mélanie Cadieux, Daphné Albert, Martin Filion

**Affiliations:** a Saint-Jean-sur-Richelieu Research and Development Centre, Agriculture and Agri-Food Canada, Saint-Jean-sur-Richelieu, Quebec, Canada; University of Arizona

## Abstract

Numerous bacterial strains from the Burkholderia cepacia complex display biocontrol activity. Here, we report the complete genome sequences of five *Burkholderia* strains isolated from soil. Biosynthetic gene clusters responsible for the production of antimicrobial compounds were found in the genome of these strains, which display biocontrol activity against various lettuce pathogens.

## ANNOUNCEMENT

The Burkholderia cepacia complex is a diverse group of microorganisms (1) that includes several strains with biocontrol activity against various plant pathogens (2, 3). This group also encompasses clinical isolates capable of causing life-threatening lung infection in patients with cystic fibrosis (4). Unfortunately, these two categories of isolates are not clearly separated, and several species include both clinical and environmental isolates (5–7).

Here, five strains from the Burkholderia cepacia complex were isolated in 2019 from agricultural soils located in the Montérégie region (Quebec, Canada). Briefly, soil samples were collected as close as possible from the root system of various vegetable crop species. Samples were stored at 4°C. One gram of soil was mixed with 100 mL of a saline solution (0.9% NaCl), and the suspension was agitated for 10 min at 250 rpm. The suspension was serially diluted and plated on King’s B agar (8) supplemented with cycloheximide (100 µg mL^−1^), ampicillin (40 µg mL^−1^), and chloramphenicol (13 µg mL^−1^). The plates were incubated at 25°C for 48 h. Isolated colonies were subsequently purified on King’s B agar (25°C for 48 h). All bacterial strains were conserved at −80°C in tryptic soy broth (BD Biosciences, Franklin Lakes, NJ) supplemented with 10% glycerol (vol/vol). For the genomic DNA (gDNA) extraction, cells were harvested from King’s B agar plate incubated at 25°C for 48 h, and gDNA was extracted with the DNeasy UltraClean microbial kit (Qiagen, Toronto, Ontario, Canada) according to the manufacturer’s instructions. Genomic DNA libraries were prepared using the PacBio SMRTbell Express template prep kit (Pacific Biosciences, Menlo Park, CA), and the genomes were sequenced on a PacBio Sequel sequencer (v3 chemistry) at the Integrated Microbiome Resource (Halifax, Nova Scotia, Canada), generating an average of 261,864 reads with an average length of 4,101 bp. The quality of the raw reads was checked with FastQC v0.11.9 (9). Genome assembly was performed using the long-read assembler Flye v2.8.1 (10), generating three circular replicons for each strain, as well as a 216-kb plasmid for strain B21-006. Default parameters were used for all software unless otherwise specified. The genomes were annotated with the NCBI Prokaryotic Genome Annotation Pipeline v5.3 (11).

**FIG 1:**
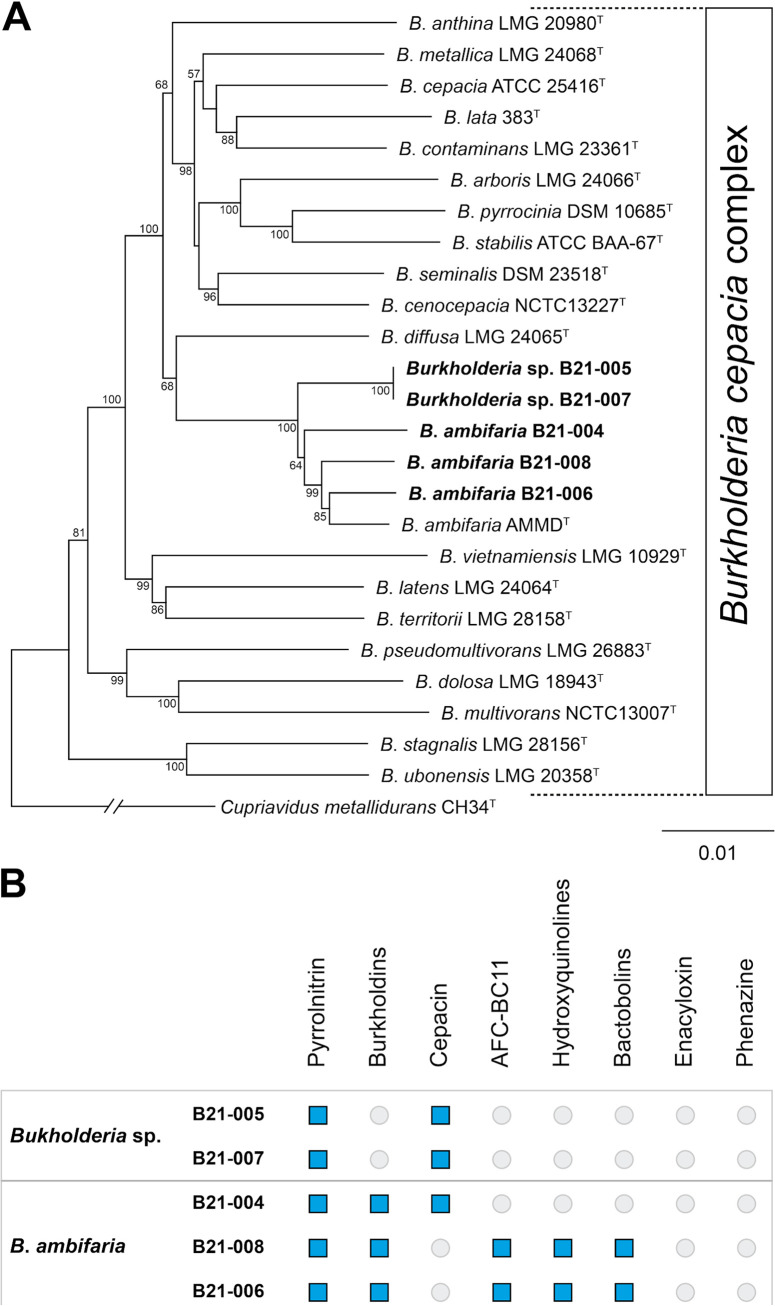
Phylogeny of the Burkholderia cepacia complex and distribution of biocontrol-related traits in the five sequenced strains. (A) Neighbor-joining phylogeny of the Burkholderia cepacia complex based on the concatenated alignment of seven housekeeping genes (*atpD*, *gltB*, *gyrB*, *lepA*, *phaC*, *recA*, and *trpB*). The complete sequences of these seven genes were concatenated and aligned using MUSCLE (16), and the phylogenetic tree was generated using the Geneious tree builder (Biomatters, Auckland, New Zealand) with the Jukes-Cantor method. Strains whose genomes are reported in this study are highlighted in bold. Only bootstrap values above 50 (from 1,000 replicates) are shown. Cupriavidus metallidurans CH34^T^ was used as an outgroup. (B) Distribution of various biocontrol-related biosynthetic gene clusters in the genomes of the five *Burkholderia* strains.

Multilocus sequence analysis with seven housekeeping genes (*atpD*, *gltB*, *gyrB*, *lepA*, *phaC*, *recA*, and *trpB*) was performed to understand the phylogenetic relationships between the five sequenced strains and the type strains of the twenty species constituting the B. cepacia complex (Fig. 1A). The five strains clustered with Burkholderia ambifaria AMMD^T^. Species-level identification of the five strains was performed using digital DNA-DNA hybridization values provided by the Type (Strain) Genome Server (12). Three strains (B21-004, B21-008, and B21-006) were shown to belong to the species B. ambifaria, whereas the two other strains (B21-005 and B21-007) were found to be just outside the species boundary of B. ambifaria.

The five *Burkholderia* strains were shown to display biocontrol activity against various lettuce pathogens under *in vitro* conditions, including Pseudomonas cichorii, Xanthomonas campestris, Pectobacterium carotovorum, and Sclerotinia sclerotiorum. Thus, we searched for biosynthetic gene clusters (BCGs) responsible for the production of antimicrobial compounds in the five genomes. Each strain harbors the BCG responsible for the biosynthesis of pyrrolnitrin, a secondary metabolite with antimicrobial activity (13). In addition, several strains harbor the BCG responsible for the production of burkholdins, cepacin, AFC-BC11, hydroquinolinines, and bactobolins (Fig. 1B). These BCGs were recently found to be present in various B. ambifaria strains with biocontrol activity (2). The BCGs responsible for the production of phenazine and enacyloxin are not present in any of the five strains, despite them being present in numerous strains from the B. cepacia complex (2, 14, 15).

### Data availability.

The complete genomes of the five *Burkholderia* strains have been deposited at DDBJ/ENA/GenBank. The raw sequencing data have been deposited into the Sequence Read Archive (BioProject accession number PRJNA775892). The accession numbers are provided in Table 1. The versions described in this paper are the first versions.

**TABLE 1:** Genomic features of the five sequenced *Burkholderia* strains

Characteristic	Data for:				
	B21-004	B21-005	B21-006	B21-007	B21-008
Genome size (Mb)	7.49	7.72	7.61	7.8	7.43
GC content (%)	66.7	66.5	66.6	66.5	66.7
Coverage (×)	144	159	110	136	51
No. of reads	257,869	417,320	215,896	284,196	134,037
Avg read length (bp)	4,738	3,392	4,486	4,300	3,592
No. of CDSs^*a*^	6,692	6,961	6,729	7,050	6,575
No. of pseudogenes	94	141	92	142	213
No. of rRNAs	18	18	18	18	18
No. of tRNAs	68	67	68	68	67
GenBank accession no.	CP086304, CP086305, CP086306	CP086301, CP086302, CP086303	CP086297, CP086298, CP086299, CP086300	CP086294, CP086295, CP086296	CP086291, CP086292, CP086293
SRA accession no.	SRR16676986	SRR16676985	SRR16676984	SRR16676988	SRR16676987

a CDSs, coding DNA sequences.
